# Affective temperament and emotional dysregulation in cyclothymia and adult ADHD: differential characteristics and clinical implications.

**DOI:** 10.1192/j.eurpsy.2024.531

**Published:** 2024-08-27

**Authors:** M. Moriconi, D. Bartolini, U. De Rosa, M. Barbuti, E. Schiavi, G. Perugi

**Affiliations:** ^1^Clinical and Experimental Medicine, University of Pisa; ^2^UO Psichiatria 2, Azienda Ospedaliero Universitaria Pisana, Pisa, Italy

## Abstract

**Introduction:**

Emotional dysregulation is central to the problem of the overlap between attention‐deficit/hyperactivity disorder (ADHD) and cyclothymia.

**Objectives:**

We aimed to compare clinical characteristics, psychiatric comorbidity, affective temperament, and emotional dysregulation among subjects with attention-deficit/hyperactivity disorder (ADHD) and cyclothymia.

**Methods:**

In this cross-sectional study, 187 participants were consecutively recruited between January 2018 and December 2019 at the outpatient clinic of the 2^nd^ Psychiatry Unit of the University Hospital of Pisa. Eighty-one subjects were diagnosed with ADHD, 62 with cyclothymic disorder, and 44 with both conditions. Participating psychiatrists collected socio-demographic and clinical data, psychiatric comorbidities according to DSM-5 criteria, familiarity for psychiatric disorders, and any previous responses to antidepressant drug therapy. To study the temperamental characteristics of the participants, the short version of the Memphis, Pisa, Paris and San Diego Temperament Assessment (Brief-TEMPS-M) was administered, while emotional dysregulation was measured through the Reactivity, Intensity, Polarity, Stability questionnaire (RIPoSt-40).

**Results:**

Cyclothymic subjects, both with and without ADHD, were more often female (p<0.001) than subjects with ADHD. Participants with ADHD showed significantly lower educational attainment than subjects without ADHD (p<0.001). In addition, participants with ADHD alone showed comorbid substance use disorder more frequently (p<0.001) than subjects with cyclothymia alone. On the other hand, the latter showed higher rates of eating disorders (p=0.033) and familiarity for major depressive disorder (p=0.009) and panic disorder (p=0.029). Depressive and anxious temperament was significantly more represented in cyclothymic subjects without ADHD, as was negative emotionality, while hyperthymic temperament showed an opposite trend. No significant differences were observed between groups for cyclothymic temperament and overall negative emotional dysregulation, but patients comorbid with both conditions had the highest scores in these subscales.

**Conclusions:**

ADHD and cyclothymia show high and overall similar levels of emotional dysregulation. However, cyclothymic patients may be more prone to negative emotionality (“dark cyclothymia”). It is possible that individuals with “sunny” cyclothymic features may escape clinical attention if ADHD is not present in comorbidity.

**Disclosure of Interest:**

None Declared

